# Insuficiência Cardíaca com Fração de Ejeção Levemente Reduzida: Considerações Terapêuticas e Justificativas dessa Renomeação

**DOI:** 10.36660/abc.20210752

**Published:** 2022-07-07

**Authors:** Eduardo Thadeu de Oliveira Correia, Evandro Tinoco Mesquita

**Affiliations:** 1 Hospital Universitário Antônio Pedro Niterói RJ Brasil Hospital Universitário Antônio Pedro, Niterói, RJ – Brasil; 2 Complexo Hospitalar de Niterói Niterói RJ Brasil Complexo Hospitalar de Niterói, Niterói, RJ – Brasil

**Keywords:** Doenças Cardiovasculares, Insuficiência Cardíaca, Volume Sistólico, Prognóstico, Antagonistas de Receptores de Mineralocorticoides, Inibidores de Enzima Conversora de Angiotensina, Digoxina

## Introdução

A Insuficiência Cardíaca (IC) tem sido classicamente dividida em IC com fração de ejeção reduzida (ICFEr) e IC com fração de ejeção preservada (ICFEp). No entanto, para classificar melhor os pacientes com IC com fração de ejeção do ventrículo esquerdo (FEVE) entre 41 e 49%, diretrizes anteriores introduziram o termo IC com fração de ejeção intermediária (ICFEi).^1^ No entanto, logo após sua introdução formal, a ICFEi passou a ser chamada de IC com fração de ejeção levemente reduzida (ICFLER).^2^ Neste artigo, exploramos as razões por trás dessa renomeação e o motive que torna essa mudança mais importante do que parece.

### Prevalência, características e prognóstico

**Figura 1:**
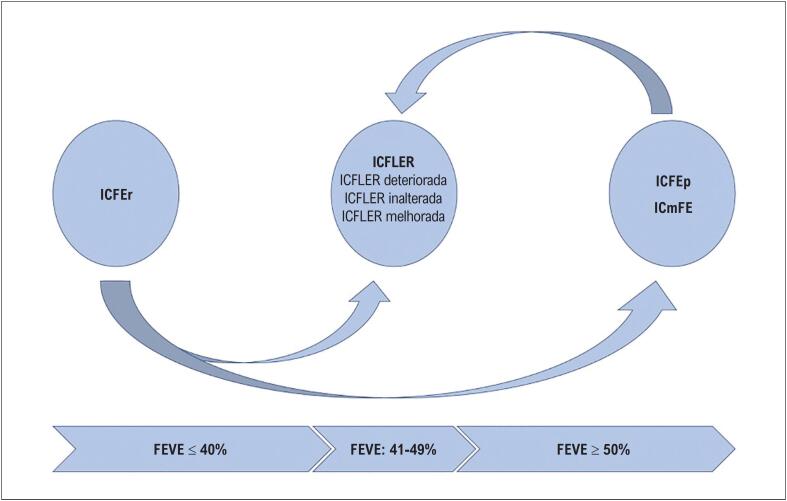
Fenótipos de Insuficiência Cardíaca de acordo com a Fração de Ejeção do Ventrículo Esquerdo. ICFLER: insuficiência cardíaca com fração de ejeção levemente reduzida; ICmFE: insuficiência cardíaca com melhora da fração de ejeção; ICFEp: insuficiência cardíaca com fração de ejeção preservada; ICFEr: insuficiência cardíaca com fração de ejeção reduzida; FEVE: fração de ejeção do ventrículo esquerdo. A ICFEr abrange pacientes com FEVE ≤ 40%. No entanto, alguns deles podem ter um aumento de 10 pontos em relação à FEVE basal e se tornar ICmFE. A ICFEr compreende FEVE de 41-49%, podendo ser pacientes com FEVE inalterada, com FEVE deteriorada e com FEVE melhorada antes de atingir os critérios de ICmFE. Por fim, os pacientes com FEVE ≥ 50% são classificados como ICFEp.

A ICFLER afeta 13-24% da população com IC.^1^ Precisamente no Brasil, 19,6% dos pacientes com IC foram classificados como ICFLER pela comunidade médica.^3^ Enquanto diretrizes anteriores indicavam que a ICFLER se assemelhava mais à ICFEp,^1^ muitas evidências publicadas desde a introdução do termo mostraram que esse grupo se assemelha mais à ICFEr ou tem características intermediárias.^1^ O prognóstico da ICFLER, por outro lado, é melhor do que o da ICFEr.^1^ É importante ressaltar que a ICFEr compreende indivíduos com diferentes trajetórias de Fração de Ejecção de Ventrículo Esquerdo (FEVE) (por exemplo, ICFEp com FEVE deteriorada; ICFEr com FEVE melhorada, ou ICFLER com FEVE inalterada), cujos prognósticos são diferentes.^1^ Isso reflete a heterogeneidade da ICFLER em comparação com a ICFEr e a ICFEp. Os fenótipos de IC de acordo com a FEVE estão descritos na Figura 1.

### Considerações terapêuticas para insuficiência cardíaca com fração de ejeção levemente reduzida

Inibidores da Enzima Conversora da Angiotensina (IECA), Bloqueadores dos Receptores da Angiotensina (BRAs) e Inibidores do Receptor da Angiotensina-Neprilisina (IRAN)

As evidências de eficácia dos BRAs para ICFEr são controversas. Em uma análise post-hoc do estudo CHARM-Preserved, a candesartana mostrou-se eficaz em comparação ao placebo para redução do desfecho de morte cardiovascular (CV) ou hospitalização por IC (HR: 0,76, IC95%: 0,61-0,96) e hospitalização por IC isolada (HR: 0,72; IC95%: 0,55-0,95).^4^ No entanto, em uma análise de desfechos pré-especificados do estudo I-PRESERVE, o irbesartan não teve efeito sobre morte por problemas cardiovasculares ou hospitalização por IC (HR: 0,98; 95%IC: 0,85-1,12) em pacientes com FEVE entre 45 e 59%.^5^

As evidências sobre o efeito de IECAs na ICFLER também são limitadas. No estudo PEP-CHF, o perindopril não teve efeito de redução da mortalidade por todas as causas, morte cardiovascular ou hospitalização por IC.^6^ No entanto, o estudo incluiu uma grande proporção de pacientes com ICFEp.

Em relação aos IRAN, em uma análise pré-especificada do estudo PARAGON-HF, a combinação sacubitril/valsartana reduziu significativamente os casos de morte cardiovascular ou hospitalização por IC em comparação com apenas valsartana em pacientes com FEVE <57%.^7^ Uma análise post-hoc adicional, que combinou dados dos estudos PARAGON-HF e PARADIGM-HF, mostrou uma redução significativa do risco no desfecho composto de hospitalização por IC ou morte cardiovascular em indivíduos com ICFEr e ICFLER.^8^ Por esse motivo, o Food and Drug Administration (FDA) expandiu as indicações na bula de sacubitril/valsartana, incluindo ICFEr e ICFLER. Assim, embora essa evidência apenas gere hipóteses, os pacientes com ICFLER provavelmente se beneficiam de sacubitril/valsartana.

### Antagonistas dos Receptores de Mineralocorticoides (ARM)

Uma análise post-hoc do estudo TOPCAT mostrou que, embora a espironolactona traga mais benefícios em casos de FEVE mais baixa, ela não melhorou os resultados em pacientes com FEVE entre 44 e 50%.^9^ No entanto, uma diferença regional significativa foi observada. Enquanto os pacientes das Américas tiveram uma redução significativa de 18% no risco de desfecho primário, na Rússia e na Geórgia, a espironolactona não melhorou o prognóstico.^10^ Análises adicionais mostraram uma proporção substancial de pacientes da Rússia e da Geórgia que não receberam ou tomaram espironolactona,^11^ o que pode explicar essa diferença. Além disso, dados de uma meta-análise que incluiu 11 ensaios clínicos randomizados (ECRs) mostrou que a espironolactona reduziu significativamente o risco de hospitalizações, melhorou a classe funcional da New York Heart Association e diminuiu os níveis de peptídeo natriurético tipo B em pacientes com ICFLER e ICFEp.^12^ Assim, a espironolactona é provavelmente eficaz para ICFLER.

### Inibidores do cotransportador sódio-glicose 2 (SGLT2)

No estudo EMPEROR-PRESERVED, a empagliflozina reduziu significativamente o risco combinado de morte cardiovascular ou hospitalização por IC em comparação com placebo em pacientes com FEVE >40%, embora esse benefício tenha vindo da redução nas hospitalizações por IC.^13^ Em uma análise de subgrupo pré-especificada, a empagliflozina foi ainda mais eficaz para a ICFLER e reduziu significativamente o risco do desfecho composto em 29% da amostra em comparação com placebo.^13^

### Betabloqueadores e Digoxina

Em uma metanálise de dados de pacientes individuais, os betabloqueadores reduziram o risco de mortalidade cardiovascular em pacientes com ICFLER em ritmo sinusal, mas não melhoraram os desfechos de pacientes com ICFLER com fibrilação atrial (FA).^14^ A digoxina, por sua vez, não melhorou o prognóstico em um análise post-hoc do estudo DIG, com pacientes com ICFLER.^15^ Os ensaios clínicos que investigaram o efeito de terapias medicamentosas para ICFEr estão descritos na Tabela 1.

**Tabela 1:** Ensaios clínicos descrevendo o efeito de terapias medicamentosas na insuficiência cardíaca com fração de ejeção levemente reduzida

Estudo	Medicação	Metodologia	Intervalo FEVE para o efeito	Mortalidade por todas as causas	mortalidade CV	Morte CV ou internação por IC	Internação por IC
PEP-CHF^6^	Perindopril	Ensaio randomizado	> 45%	1,09 (0,75-1,58)	0,98 (0,63-1,53)	NR	0,86 (0,61-1,20)
CHARM^4^	Candesartana	Análise post-hoc de um estudo randomizado	40-49%	0,79 (0,60-1,04)	0,81 (0,60-1,11)	0,76 (0,61-0,96)	0,72 (0,55-0,95)
I-PRESERVE^5^	Irbesartana	Ensaio randomizado	45-59%	NR	NR	0,98 (0,85-1,12)	NR
PARAGON-HF^7,8^	Sacubitril-Valsartana	Ensaio randomizado	45-50%	NR	NR	0,82 (0,63–1,06)	NR
TOPCAT^9,10^	Espironolactona	Análise post-hoc de um estudo randomizado	44-50%	0,73 (0,49-1,10)	0,69 (0,43-1,12)	0,72 (0,50-1,05)	0,76 (0,46-1,27)
Xiang et al.^12^	Espironolactona	Metanálise de estudos randomizados	> 40%	NR	0,72 (0,31–1,69)	NR	0,84 (0,73–0,95)
Cleland et al.^14^	Bloqueadores beta	Metanálise de dados de pacientes individuais	40-49%	SR: 0,59 (0,34-1,03); AF: 1,30 (0,63-2,67)	SR: 0,48 (0,24-0,97); AF: 0,86 (0,36-2,03)	SR: 0,83 (0,60-1,13); AF: 1,06 (0,58-1,94)	SR: 0,95 (0,68-1,32); AF: 1,15 (0,57-2,32)
EMPEROR-Preserved^13^	Empagliflozina	Ensaio randomizado	> 40%	1,00 (0,87-1,15)	0,91 (0,76-1,09)	0,79 (0,69-0,90)	0,73 (0,61-0,88)
DIG^15^	Digoxina	Análise post-hoc de um estudo randomizado	40-49%	1,08 (0,85-1,37)	1,24 (0,94-1,64)	0,96 (0,79-1,17)	0,80 (0,63-1,03)

CV: cardiovascular; IC: insuficiência cardíaca; FEVE: fração de ejeção do ventrículo esquerdo; NR: não reportado.

### Necessidades atuais

Diretrizes anteriores sugeriram que pacientes com ICFLER deveriam ser tratados como ICFEp. No entanto, como mencionado anteriormente, esses pacientes se beneficiam de múltiplas terapias das quais os pacientes com ICFEp não tiram proveito. Além disso, a ICFLER é semelhante à ICFEr. Futuros ECRs devem alocar pacientes com ICFLER para que as recomendações possam ser estendidas a esse grupo. Isso poderia ser feito pela inclusão da ICFLER em estudos sobre ICFEr ou por meio de estudos específicos para essa população, embora seja uma alternativa desafiadora.

## Conclusões

A ICFEi se assemelha principalmente à ICFEr e se beneficia de várias terapias. A transição de seu antigo nome para ICFLER é apropriada e dá a sensação de que esses pacientes se beneficiam das terapias de ICFEr. Isso pode levar a um aumento na adoção de terapias baseadas em diretrizes, melhorando os resultados nesse grupo de pacientes historicamente esquecido.
