# Supplementary material for: Insuficiência Cardíaca com Fração de Ejeção Levemente Reduzida: Considerações Terapêuticas e Justificativas dessa Renomeação
Source: Arq Bras Cardiol. 2022 Jul 7;119(1):124–7. [Article in Portuguese] doi: 10.36660/abc.20210752 (PMC9352121; doi:10.36660/abc.20210752)
Supplement: Translation

# Translation: Heart Failure with Mildly Reduced Ejection Fraction: Therapeutical Considerations and Reasons for This Renaming

**Authors:** Eduardo Thadeu de Oliveira Correia, Evandro Tinoco Mesquita

**Affiliations:** 1 Niterói RJ Brazil Hospital Universitário Antônio Pedro, Niterói, RJ – Brazil; 2 Niterói RJ Brazil Complexo Hospitalar de Niterói, Niterói, RJ – Brazil

**Keywords:** Cardiovascular Diseases, Heart Failure, Stroke Volume, Prognosis, Mineralocortical Receptor Antagonists, Angiotensin Converting Enzyme Inhibitors, Digoxin

## Introduction

Heart failure (HF) has been classically divided into HF with reduced ejection fraction (HFrEF) and HF with preserved ejection fraction (HFpEF). However, to better classify HF patients with a left ventricular ejection fraction (LVEF) between 41 and 49%, previous guidelines have introduced the term HF with mid-range ejection fraction.^1^ Nonetheless, shortly after its formal introduction, HF with mid-range ejection fraction is now called HF with mildly reduced ejection fraction (HFmrEF).^2^ In this letter, we explore the reasons behind this renaming and why this change is more important than it may seem.

### Prevalence, Characteristics and Prognosis

HFmrEF comprises 13-24% of the HF population.^1^ Specifically in Brazil, 19.6% of HF patients were classified as HFmrEF in the community.^3^ While previous guidelines indicated that HFmrEF resembled more HFpEF,^1^ extensive evidence published since its introduction showed this group is more similar to HFrEF or have intermediate characteristics.^1^ On the other hand, prognostically, HFmrEF has better outcomes than HFrEF.^1^ Importantly, HFmrEF comprehends individuals with different LVEF trajectories (e.g. HFpEF with a deteriorated LVEF; HFrEF with an improved LVEF or HFmrEF with an unchanged LVEF) that have different prognosis.^1^ This highlights the heterogeneity of HFmrEF compared with HFrEF and HFpEF. HF phenotypes according to LVEF are described in Figure 1.

**Figure 1:** Heart Failure Phenotypes according to Left Ventricular Ejection Fraction. HFmrEF – heart failure with mildly reduced ejection fraction; HFimpEF – heart failure with improved ejection fraction; HFpEF: heart failure with preserved ejection fraction; HFrEF: heart failure with reduced ejection fraction; LVEF> left ventricular ejection fraction. HFrEF covers patients with a LVEF ≤ 40%. Nonetheless, some of these patients can have a 10-point increase from baseline LVEF and become HFimpEF. HFmrEF comprises patients with LVEF from 41-49%, which could be patients with an unchanged LVEF; patients with a deteriorate LVEF and patients with an improved LVEF before reaching HFimpEF criteria. Finally, patients with a LVEF ≥ 50% are classified as HFpEF.

### Therapeutical Considerations for Heart Failure with Mildly Reduced Ejection Fraction

Angiotensin-converting-enzyme inhibitors (ACEi), Angiotensin receptor blockers (ARBs) and Angiotensin Receptor-Neprilysin Inhibitors (ARNI)

Evidence for the effectiveness of ARBs in HFmrEF is controversial. In a post-hoc analysis of the CHARM-Preserved trial, candesartan was shown to be effective compared to placebo in reducing the composite end-point of cardiovascular (CV) death or HF hospitalization (HR: 0.76, 95%CI: 0.61-0.96) and HF hospitalization alone (HR: 0.72; 95%CI: 0.55-0.95).^4^ However, in a prespecified analysis of the I-PRESERVE trial, irbesartan had no effect on CV death or HF hospitalization (HR: 0.98; 95%CI: 0.85-1.12) in patients with a LVEF between 45 and 59%.^5^ Evidence on the effect of ACEi in HFmrEF is limited. In the PEP-CHF trial observed perindopril had no effect on reducing all-cause mortality, CV death or HF hospitalization.^6^ Nevertheless, this trial included a large proportion of HFpEF patients. Regarding ARNI, in a prespecified analysis of the PARAGON-HF trial, sacubitril/valsartan significantly reduced CV death or HF hospitalization compared with valsartan alone in patients with a LVEF <57%.^7^ A further post-hoc analysis that combined data from the PARAGON-HF and PARADIGM-HF trials, showed that individuals with HFrEF and HFmrEF had a significant risk reduction in the composite endpoint of HF hospitalization or CV death.^8^ For this reason, the FDA extended the indication of sacubitril/valsartan in the package insert to include HFrEF and HFmrEF. Therefore, although this evidence is hypothesis-generating only, patients with HFmrEF probably benefit from sacubitril/valsartan.

### Mineralocorticoid receptor antagonists (MRA)

A post-hoc analysis of the TOPCAT trial showed that, although spironolactone had greater benefits at lower LVEF, it did not improve outcomes in patients with LVEF between 44 and 50%.^9^ Nonetheless, a significant regional difference was observed in the TOPCAT trial. While patients enrolled in the Americas had a significant 18% risk reduction in the primary outcome, in Russia and Georgia, spironolactone did not improve prognosis.^10^ Further analysis showed that a substantial proportion of patients enrolled in Russia and Georgia did not receive or take spironolactone,^11^ which can explain this difference. Also, data from a meta-analysis that included 11 randomized controlled trials (RCTs) showed spironolactone significantly reduced the risk of hospitalizations, improved New York Heart Association functional class and decreased levels of b-type natriuretic peptide in HFmrEF and HFpEF patients.^12^ Thus, spironolactone is probably effective in HFmrEF.

### Sodium–glucose cotransporter 2 inhibitors

In the EMPEROR-PRESERVED trial, empagliflozin significantly reduced the combined risk of CV death or HF hospitalization compared with placebo in patients with a LVEF >40%, although this benefit came from the reduction in HF hospitalizations.^13^ In a prespecified subgroup analysis, empagliflozin was even more effective in HFmrEF, and significantly reduced the risk of the composite outcome by 29% compared with placebo.^13^

### Beta-blockers and Digoxin

In an individual patient data meta-analysis, beta-blockers reduced the risk of CV mortality in HFmrEF patients in sinus rhythm, but did not improve endpoints in HFmrEF patients with AF.^14^ Digoxin, on the other hand, did not improve prognosis in a post-hoc analysis of the DIG trial for HFmrEF patients.^15^ Clinical Trials that investigated the effect of drug therapies for HFmrEF are described in Table 1.

**Table 1:** Clinical trials describing the effect of drug therapies in Heart Failure with Mildly Reduced Ejection Fraction

Study	Drug	Methodology	LVEF range for the Effect	All-Cause Mortality	CV Mortality	CV Death or HF Hospitalization	HF Hospitalization
PEP-CHF^6^	Perindopril	Randomized Trial	> 45%	1.09 (0.75-1.58)	0.98 (0.63-1.53)	NR	0.86 (0.61-1.20)
CHARM^4^	Candesartan	Post-hoc analysis of a randomized trial	40-49%	0.79 (0.60-1.04)	0.81 (0.60-1.11)	0.76 (0.61-0.96)	0.72 (0.55-0.95)
I-PRESERVE^5^	Irbesartan	Randomized Trial	45-59%	NR	NR	0.98 (0.85-1.12)	NR
PARAGON-HF^7,8^	Sacubitril-Valsartan	Randomized Trial	45-50%	NR	NR	0.82 (0.63–1.06)	NR
TOPCAT^9,10^	Spironolactone	Post-hoc analysis of a randomized trial	44-50%	0.73 (0.49-1.10)	0.69 (0.43-1.12)	0.72 (0.50-1.05)	0.76 (0.46-1.27)
Xiang et al.^12^	Spironolactone	Meta-analysis of randomized trials	> 40%	NR	0.72 (0.31–1.69)	NR	0.84 (0.73–0.95)
Cleland et al.^14^	Beta-blockers	Meta-analysis of individual patient data	40-49%	SR: 0.59 (0.34-1.03); AF: 1.30 (0.63-2.67)	SR: 0.48 (0.24-0.97); AF: 0.86 (0.36-2.03)	SR: 0.83 (0.60-1.13); AF: 1.06 (0.58-1.94)	SR: 0.95 (0.68-1.32); AF: 1.15 (0.57-2.32)
EMPEROR-Preserved^13^	Empagliflozin	Randomized Trial	> 40%	1.00 (0.87-1.15)	0.91 (0.76-1.09)	0.79 (0.69-0.90)	0.73 (0.61-0.88)
DIG^15^	Digoxin	Post-hoc analysis of a randomized trial	40-49%	1.08 (0.85-1.37)	1.24 (0.94-1.64)	0.96 (0.79-1.17)	0.80 (0.63-1.03)

AF: atrial fibrillation; CV: cardiovascular; HF: heart failure; LVEF: left ventricular ejection fraction; SR: sinus rhythm.

### Current needs

Previous guidelines suggested HFmrEF patients should be treated as HFpEF. However, as previously mentioned, these patients benefit from multiple therapies that HFpEF patients do not. In addition, as seen, HFmrEF is similar to HFrEF. Future RCTs should randomize HFmrEF patients so guideline recommendations can be extended to this group. This could be accomplished through the inclusion of HFmrEF in HFrEF trials or by conducting trials specifically for this population, although this is a challenging alternative.

## Conclusions

HFmrEF mostly resembles HFrEF and benefits from multiple therapies. The transition from its former name HF with mid-range ejection fraction to HFmrEF is appropriate and gives the sense that these patients benefit from HFrEF therapies. This may lead to an increase in the adoption of guideline-directed medical therapies, improving outcomes in this historically forgotten group of patients.
